# Malignant solitary fibrous tumor of the seminal vesicle: a case report and review of the literature

**DOI:** 10.1186/s12894-025-01815-6

**Published:** 2025-05-10

**Authors:** Xuerong Ye, Hao Yu, Bo Sun, Jiakuan Li, Jiage Sun, Bei Hou, Shan Liu, Xiaoyi Zhang

**Affiliations:** 1 Department of Urology, The Characteristic Medical Center of PLA Rocket Force, NO.16 Xinjiekouwai Street, Beijing, 100088 China; 2 Department of Geriatric Medicine, Electric Power Teaching Hospital of Capital Medical University, Beijing, 100073 China; 3 Department of Urology, Strategic Support Force Characteristic Medical Center of PLA, Beijing, 100101 China; 4 Department of Pathology, The Characteristic Medical Center of PLA Rocket Force, Beijing, 100088 China

**Keywords:** Case report, Solitary fibrous tumor (SFT), Seminal vesicle tumor, Surgery

## Abstract

**Background:**

Solitary fibrous tumors (SFTs) are rare mesenchymal tumors that can occur in multiple parts of the human body. The majority of SFTs are benign, with malignant cases being exceedingly rare. Although SFTs have been identified in extrapleural sites such as the upper respiratory tract, orbits, and extremities, their occurrence in the seminal vesicles is exceedingly uncommon. To date, only a few cases of seminal vesicle SFTs have been documented, making this case notable for its rarity and clinical presentation.

**Case presentation:**

A 43-year-old male patient was incidentally found to have a left seminal vesicle mass on an MRI scan during a routine health check-up. A subsequent PET‒CT scan revealed enlargement of the left seminal vesicle with uneven density and FDG uptake, raising suspicion of malignancy. Although a biopsy suggested a solitary fibrous tumor of the seminal vesicle, the limited tissue sample prevented definitive exclusion of malignancy. This highlights the diagnostic challenges of such rare tumors, particularly when biopsy samples are insufficient. To address this, rapid intraoperative pathology was employed, which confirmed the malignancy and informed the patient of the subsequent surgical approach. The patient underwent laparoscopic excision of the left seminal vesicle tumor, followed by radical excision of both the prostate and seminal vesicles. Postoperatively, the patient recovered well, and final pathology confirmed a malignant solitary fibrous tumor. After five years of follow-up, the patient remained free from recurrence or metastasis.

**Conclusion:**

Although the preoperative biopsy in this case established the diagnosis of SFT, it did not definitively ascertain whether it was benign or malignant. Hence, intraoperative frozen section pathology plays a critical role in determining the surgical strategy. This case indicates that satisfactory therapeutic outcomes for seminal vesicle SFTs can be achieved through complete resection via minimally invasive laparoscopic surgery.

## Background

Primary malignant tumors of the seminal vesicles are extremely rare [1], with approximately 100 cases reported globally [2]. Solitary fibrous tumors originating in the seminal vesicles are even rarer [3], with approximately 9 cases reported in the English literature (4 of which were malignant). These tumors present significant diagnostic and therapeutic challenges. While most SFTs are benign, malignant transformation has been observed, requiring a comprehensive approach to diagnosis and treatment. In our case, we aimed to develop a comprehensive diagnostic and surgical strategy that would ensure accurate identification of the malignancy and effective treatment. This approach includes advanced imaging techniques, rapid intraoperative pathology, and tailored surgical intervention to achieve the best possible outcome for the patient.

## Case presentation

A 43-year-old male patient presented with an incidental finding of a left seminal vesicle mass during a routine physical examination. The patient had no preexisting medical conditions, no family history of malignant tumors, and no abnormalities on general or systemic examination. Laboratory evaluations revealed normal serum tumor marker levels (carcinoembryonic antigen: 0.75 ng/mL; carbohydrate antigen 19–9: 13.3 U/mL; alpha-fetoprotein: 2.87 ng/mL; prostate-specific antigen: 2.95 ng/mL), and routine blood and urine analyses revealed no abnormalities.

**Fig. 1:**
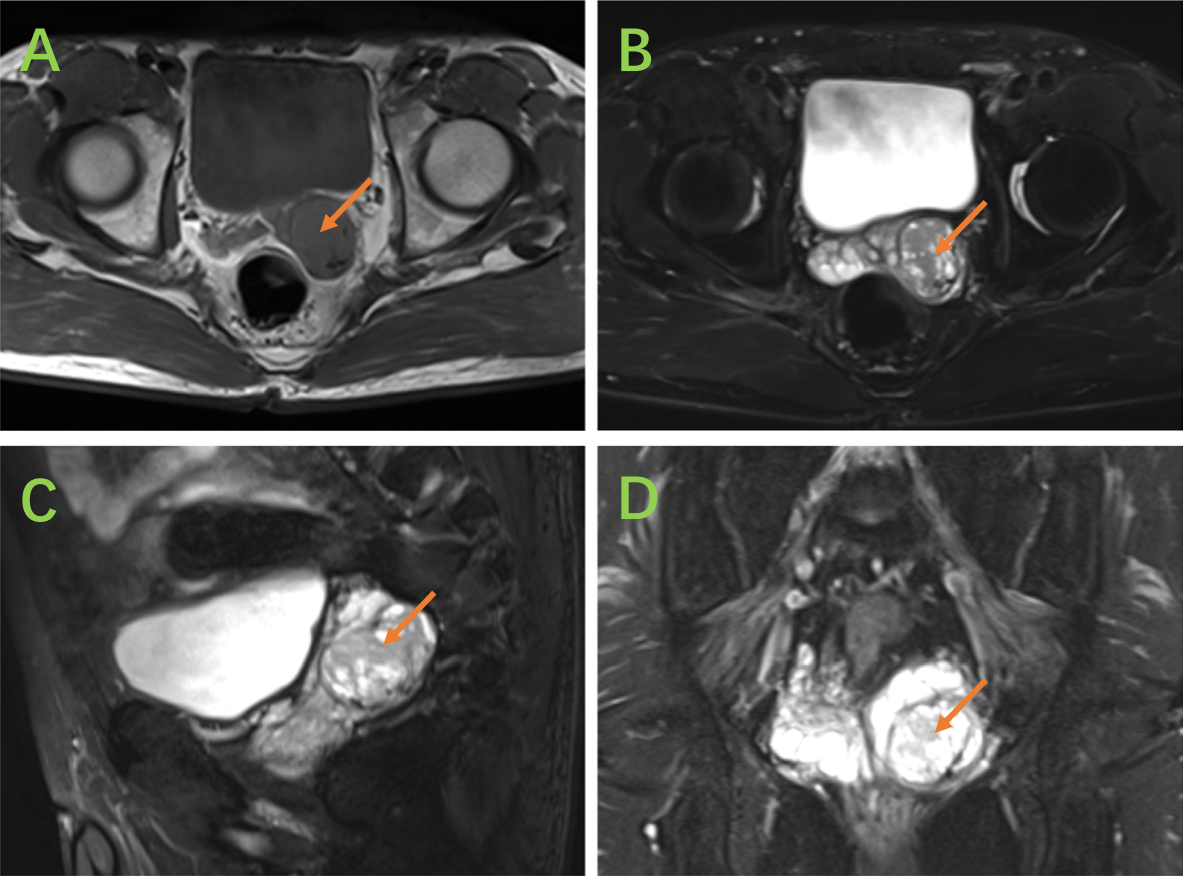
**A **Axial magnetic resonance imaging (MRI) in the T1 phase;** B **Axial MR image in the T2 phase; **C **Sagittal MR image in the T2 phase; **D **Coronal MR image in the T2 phase

**Fig. 2:**
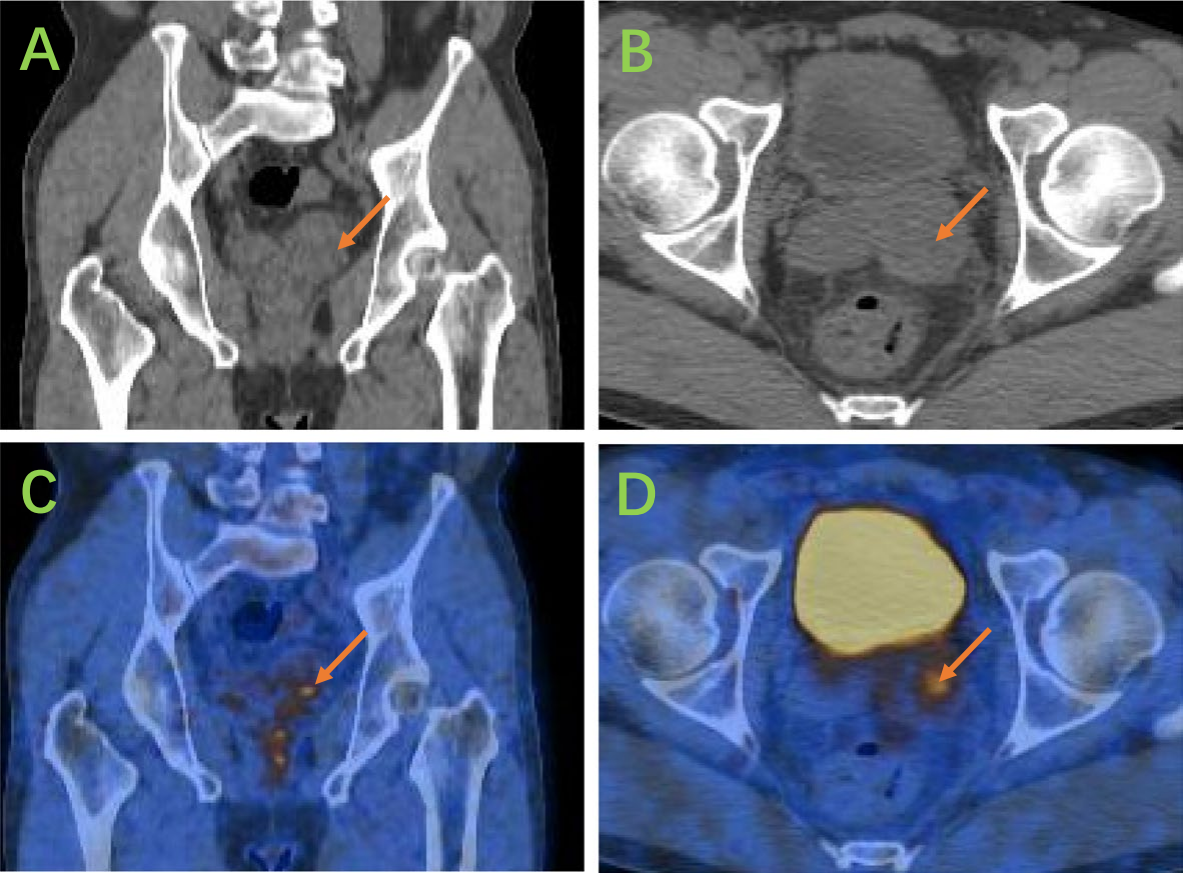
**A **The coronal section illustrates uneven density within the left seminal vesicle; **B **The axial section reveals enlargement of the left seminal vesicle; **C **An image demonstrating uptake of FDG in the left seminal vesicle; **D **An image highlighting FDG avidity (SUVmax 4.0), indicating metabolic activity

**Fig. 3:**
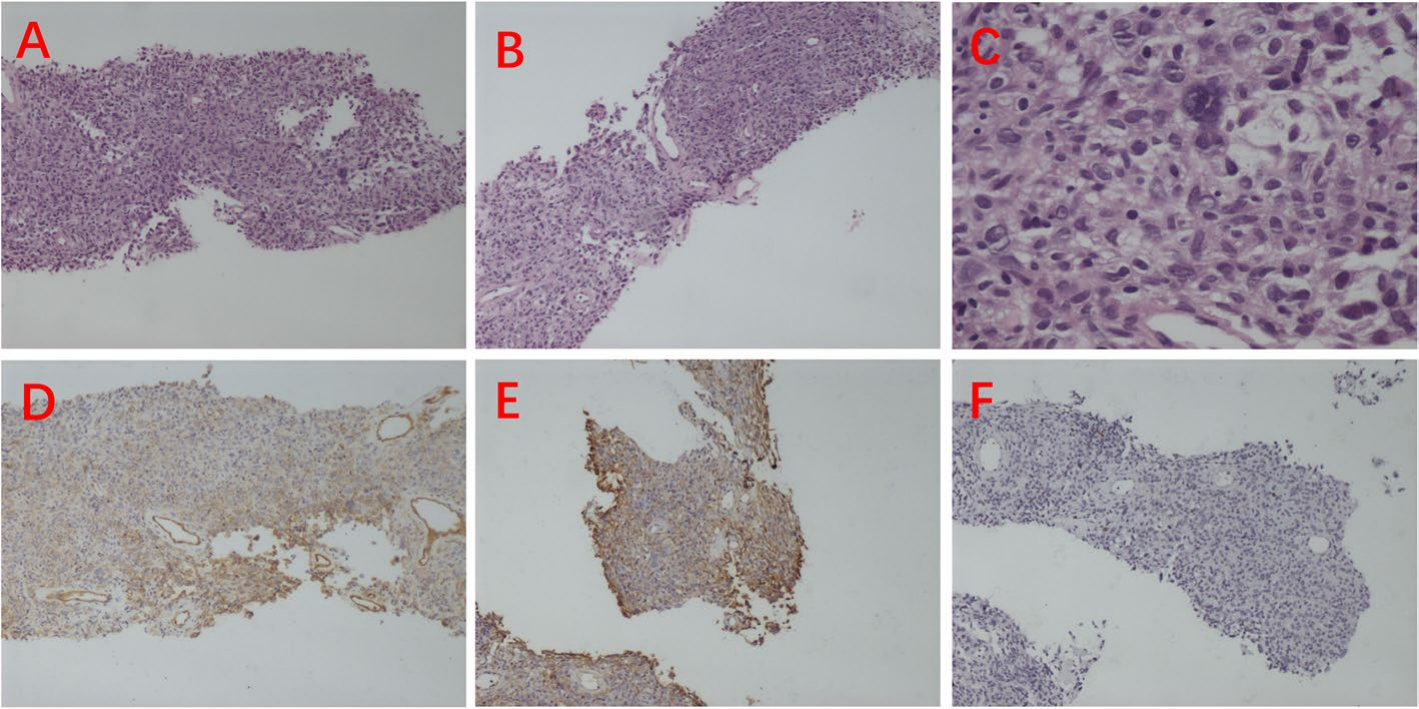
**A**, **B **Spindle-cell tumor morphology at low magnification (× 100); **C **Significant nuclear atypia with rare mitotic figures at high magnification(× 100); **D **Immunohistochemistry showing CD34(+)(× 100); **E** Immunohistochemistry showing CD99(+)(× 100); **F **Ki-67 proliferation index of 2%−10%(× 100)

Postadmission imaging with MRI revealed an enlarged left seminal vesicle characterized by a round-shaped mass with mixed equal T1 signals and long T2 signals (Fig. 1), measuring approximately 3.6 cm × 2.6 cm with a clear boundary. The mass exhibited a markedly high signal on diffusion-weighted imaging (DWI) and a decreased apparent diffusion coefficient (ADC). Additionally, septate-like short T2 signals were observed at the edges. Further diagnostic work-up via whole‒body PET‒CT revealed enlargement of the left seminal vesicle with uneven density and FDG uptake, raising suspicion of malignancy (Fig. 2). A biopsy of the mass, performed under transrectal ultrasound guidance using fine needle biopsy (FNB), suggested a solitary fibrous tumor of the seminal vesicle, which was supported by histopathological findings of spindle-shaped tumor cells with significant nuclear atypia and rare mitotic figures. The Ki-67 proliferation index was low, and immunohistochemical staining revealed positivity for CD34 and CD99 (Fig. 3). However, owing to the limited number of biopsy samples, malignancy could not be definitively excluded, and complete surgical resection was recommended for a conclusive diagnosis.

**Fig. 4:**
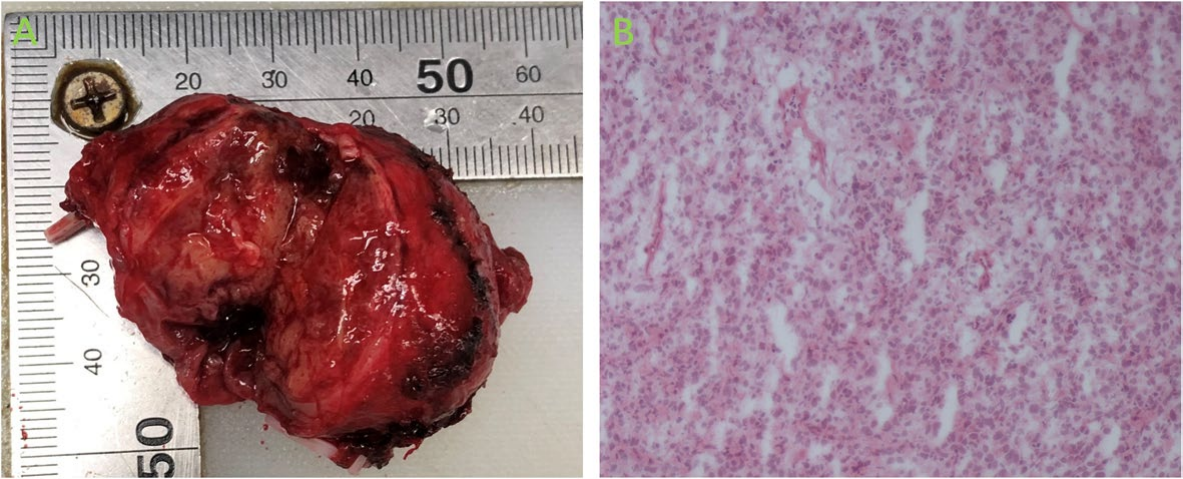
**A **Left seminal vesicle, 3.6 cm × 2.6 cm, with distinct borders; **B **Immunohistochemistry showing malignant seminal vesicle tumor(× 100)

**Fig. 5:**
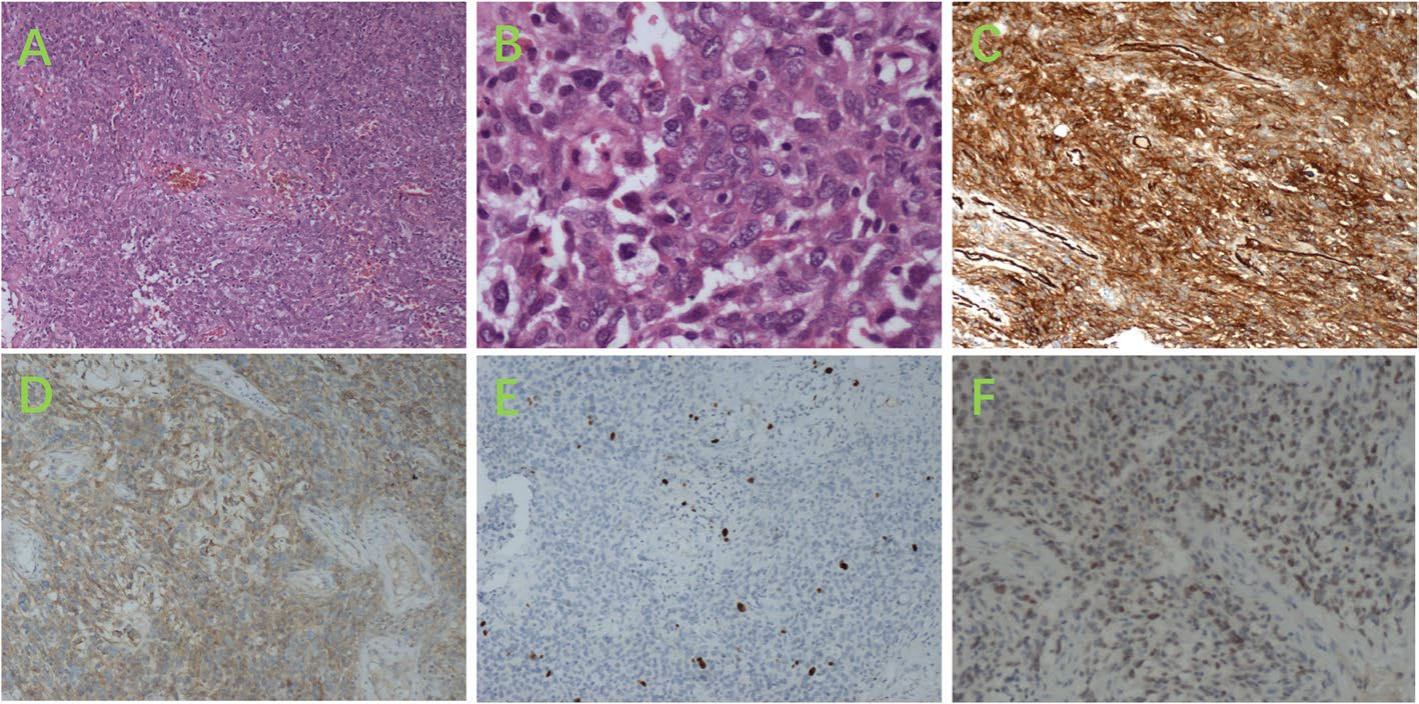
**A **HE staining showing stromal vascular proliferation with a branching pattern and hyaline degeneration of the vascular walls (× 100); **B **Significant cellular atypia with 5 mitotic figures per 10 high-power fields (× 400); **C **Immunohistochemistry showing CD34(+) (× 100); **D **Immunohistochemistry showing CD99(+)(× 100); **E **Ki-67 proliferation index of 2%−10% (× 100); **F **Immunohistochemistry showing STAT6(+)(× 100)

The patient underwent laparoscopic left seminal vesiculectomy. During surgery, the left seminal vesicle was found to have a firm texture (Fig. 4A). The mass was successfully isolated and completely excised, and the tissue was sent for pathological examination. Approximately 30 min later, the rapid pathology report identified the mass as a spindle-cell tumor with significant atypia, which was consistent with a malignant neoplasm (Fig. 4B). On the basis of these findings, radical prostatectomy was subsequently performed to excise both the prostate and seminal vesicles. Postoperative pathological examination revealed dense, short, spindle-shaped, oval tumor cells with noticeable atypia and prominent nucleoli. Approximately five mitotic figures per 10 high-power fields were observed. The tumor exhibited stromal vascular proliferation with branching patterns and hyaline degeneration of the vascular walls, indicative of malignant behavior, along with invasion into the seminal vesicle gland tissue. The surgical margins were confirmed to be clear. Immunohistochemical analysis demonstrated positivity for CD34, CD99, Ki67 (2%−10%), and STAT6 (Fig. 5), and these findings were consistent with the diagnosis of a malignant solitary fibrous tumor.

Postoperatively, the patient had a smooth recovery without urinary incontinence. However, erectile dysfunction persisted following radical prostatectomy, significantly affecting the patient's sexual function. The patient did not pursue further treatment for erectile dysfunction during the follow-up period. The patient was scheduled for follow-up MRI scans every 3–6 months during the first year postoperatively, with annual scans thereafter. Additionally, annual telephone follow-up was conducted to monitor for signs of recurrence, metastasis, and to evaluate the patient's urinary function, including incontinence.

## Discussion

Seminal vesicle malignancies are predominantly secondary and are metastases of tumors of adjacent organs; primary seminal vesicle malignancies are exceedingly rare [3]. These primary tumors can be categorized on the basis of their tissue of origin into epithelial (e.g., papillary adenoma, adenocarcinoma), mesenchymal (e.g., leiomyosarcoma, fibrosarcoma, angiosarcoma), mixed epithelial and mesenchymal (e.g., phyllodes sarcoma), and other types, which include neuroendocrine tumors, malignant ganglions, and germ cell tumors [4]. Among these, adenocarcinoma of epithelial origin is the most prevalent, albeit with fewer than 100 cases documented globally [2]. Solitary fibrous tumors (SFTs), categorized as mesenchymal tumors, are particularly rare in the seminal vesicle. The limited number of reported cases, along with occasional malignant transformation, presents significant diagnostic challenges, as these tumors often exhibit nonspecific clinical symptoms and overlapping imaging features [5].

There is no uniform pattern in the clinical manifestations of seminal vesicle SFTs, as detailed in Table 1. The characteristics of seminal vesicle SFTs, as observed in 9 previously reported cases and the present case, are summarized. The patients'ages ranged from 43 to 66 years, with an average age of 54 years. Most reported cases involve the right seminal vesicle, with only two cases affecting the left [5]. At the time of diagnosis, some patients present symptoms such as hematuria, frequent urination, dysuria, hematospermia, and pain or discomfort in the lower abdomen or groin area. Remarkably, approximately half of the patients were asymptomatic.

**Table 1:** Clinical data of the 9 reported patients and our patient with primary solitary fibrous tumors of the seminal vesicle

Case	Age	Year	Presentation	Side	Tumor Size(cm)	Histopathological features	Treatment	Follow-up(months)	Recurrence
1	47	2000	Groin discomfort	RSV	4–11	Malignant	Excision	NED(15)	None
2	46	2000	Haematospermia	RSV	4–11	Malignant	Excision	NED(8)	None
3	50	2003	An asymptomatic pelvic lump	LSV	10	Benign	Pelvic exenteration	NED(24)	None
4	65	2006	Haematuria, dysuria, frequency, and urgency	RSV	9	Benign	Excision of the tumor, distal ureter	NED(14)	None
5	56	2010	An asymptomatic pelvic lump	RSV	8	Benign	Excision	NED(8)	None
6	52	2013	An asymptomatic pelvic lump	RSV	NA	Malignant	Radiotherapy, and excision of SFT	NED(14)	None
7	66	2019	An asymptomatic pelvic lump	RSV	12	Benign	Excision	NED(6)	None
8	58	2021	Frequent micturition and lower abdominal pain	RSV	10	Malignant	Excision	NA	NA
9	54	2023	Haematuria	RSV	6	Benign	Excision	NED(24)	None
10	43	2024	An asymptomatic pelvic lump	LSV	3.6	Malignant	Radical prostatectomy	NED(60)	None

*Abbreviations*: *NA* Not available, *NED* No evidence of disease, *SFT* Solitary fibrous tumor, *RSV* Right seminal vesicle, *LSV* Left seminal vesicle

Asymptomatic seminal vesicle SFTs are often incidentally discovered during imaging procedures. For tumors originating from the seminal vesicle, pelvic CT and MRI are particularly beneficial for determining the tumor's origin and extent, as well as for further evaluating whether there is potential invasion into adjacent tissues. On cross-sectional imaging, SFTs typically present with a homogeneous signal intensity on T1-weighted images and a variable signal intensity on T2-weighted images, creating a characteristic mixed black-and-white appearance [6]. PET‒CT findings, such as FDG uptake, can further raise suspicion for malignancy. However, these imaging features are not pathognomonic, necessitating further pathological examination for definitive diagnosis.

The diagnosis of SFTs is primarily based on pathological examination results. The markers most commonly expressed by these tumors include vimentin, CD34, CD99, Bcl-2, and Ki-67, which are listed in descending order on the basis of their positive rates [7]. Additionally, STAT6 is strongly and predominantly expressed in the nucleus, as opposed to in the cytoplasm, in most SFTs, and the determination of its level plays a crucial role in the differential diagnosis of SFTs [8]. Although most SFTs exhibit a benign course, a small fraction of cases (estimated to be between 11 and 22%) may display malignant behaviours, such as recurrence or metastasis postsurgery [2]. Factors suggestive of malignant potential include a large tumor size (> 5 cm), histopathological hypercellularity, the presence of necrosis, and an increased rate of mitosis [9]. In this context, preoperative biopsy provides critical insights but may be limited by sample size or representativeness. Intraoperative rapid pathology serves as an invaluable adjunct, offering real-time confirmation of malignancy and guiding immediate surgical decisions. This diagnostic workflow highlights the importance of integrating imaging, pathology, and surgical pathology for rare and challenging cases such as seminal vesicle SFTs.

Currently, the most effective treatment for seminal vesicle SFTs is complete surgical resection of the tumor [10]. The choice of surgical approach often depends on the size of the tumor, extent of local invasion, and pathological findings. Minimally invasive techniques, such as laparoscopic or robotic-assisted surgery, are increasingly favored because of their reduced morbidity and quicker recovery [11].Among the 9 reported cases of seminal vesicle SFTs, treatment strategies ranged from simple excision to more extensive surgeries, such as radical prostatectomy. For malignant cases, a comprehensive approach, including the removal of adjacent structures, is crucial to ensure negative surgical margins and minimize the risk of recurrence [12]. In certain cases, adjuvant therapies such as chemotherapy or radiotherapy have been employed, but their role remains undefined owing to limited data [13]. In our case, considering the patient's young age and the malignant nature of the tumor, a more aggressive surgical approach was chosen; therefore, the patient underwent radical prostatectomy, which included the removal of the prostate and seminal vesicles.

## Conclusions

This article presents a case of a malignant SFT of the seminal vesicle, marking the 10 th reported case of seminal vesicle SFT and the 5 th reported case of a malignant type SFT in the English literature. This case highlights the rarity of malignant SFTs of the seminal vesicle and the importance of advanced imaging, rapid intraoperative pathology, and tailored surgical strategies for achieving successful outcomes. Long-term follow-up is essential for detecting recurrence or metastasis, and the patient was monitored for 5 years without evidence of recurrence or metastasis. Future studies could focus on the role of adjuvant therapies, as well as the molecular mechanisms underlying the malignant transformation of SFTs, to better understand the pathophysiology and improve therapeutic strategies.

## Data Availability

The datasets supporting the conclusions of this study are included within the manuscript. Additional datasets are available from the corresponding author upon reasonable request.

## Abbreviations

SFT: Solitary fibrous tumor
MRI: Magnetic resonance imaging
FDG: 18-F-fluorodeoxyglucose
PET‒CT: Positron emission tomography contrast-enhanced computed tomography
